# Comparative Analysis of the International Academy of Cytology Yokohama System and the Modified Masood Scoring Index in Fine Needle Aspiration Cytology of Palpable Breast Lesions

**DOI:** 10.7759/cureus.110506

**Published:** 2026-06-09

**Authors:** Utkarsh Tripathi, Pragati Awasthi, Tina Rai

**Affiliations:** 1 Department of Pathology, Atal Bihari Vajpayee Government Medical College, Vidisha, IND

**Keywords:** breast cancer, breast cancer pathology, breast cytopathology, carcinoma in situ, fine needle aspiration cytology (fnac), modified masood scoring index, the iac yokohama system

## Abstract

Introduction: Breast cancer has surpassed cervical cancer in India, underscoring the importance of screening and early detection. Triple testing, including clinical examination, mammography, and fine needle aspiration cytology (FNAC), is crucial for assessing suspicious breast lesions. FNAC, a simple and minimally invasive method, effectively diagnoses benign and malignant lesions, aiding treatment, though overlapping morphological features can sometimes cause diagnostic errors.

Materials and methods: This retrospective study involves patients referred for FNAC to the Department of Pathology at Atal Bihari Vajpayee Government Medical College, Vidisha, from February 2023 to July 2024. Cytology slides were retrieved and reviewed by two trained cytopathologists on a single-blind basis, and two commonly used cytological grading systems, i.e., the International Academy of Cytology (IAC) Yokohama Classification and the Modified Masood Scoring Index (MMSI), were applied. Histopathology sections of available breast lesions were reviewed and correlated with cytological diagnoses.

Results: Around 174 (out of a total of 535 fine needle aspirations done) cases of breast lesions were taken into account, for which cytohistopathological examinations were done. They were categorized according to the Yokohama and MMSI systems. Statistical analysis was done using histopathology as the standard.

Discussion: The IAC Yokohama system demonstrated superior diagnostic accuracy, sensitivity, and clinical utility compared to the MMSI. The Yokohama system’s risk of malignancy (ROM)-based, five-tiered structure provides clearer management pathways and reduced ambiguity, establishing it as the preferred universal reporting system for routine breast cytopathology.

Conclusion: The Yokohama system provides superior diagnostic accuracy, standardized risk stratification, and practical clinical communication compared to the MMSI. Its globally validated, ROM-based framework makes it the preferred, more reliable reporting standard.

## Introduction

Breast cancer has emerged as the most frequently diagnosed cancer among females in India, overtaking cervical cancer in recent years. Early identification of suspicious breast lesions remains central to improving survival outcomes [1]. In routine clinical practice, the combined use of physical examination, mammography, and core needle biopsy, commonly termed the triple assessment, provides a reliable framework for evaluating breast abnormalities [1]. The gold standard for the diagnosis of breast cancer is core needle biopsy, but fine needle aspiration cytology (FNAC) might be useful for screening of suspicious lesions and in situations where biopsy is not possible. FNAC plays a key role because it is minimally invasive, cost-effective, and capable of offering rapid cytomorphological information that guides early clinical decision-making [2,3].

To standardize reporting and improve diagnostic communication, the International Academy of Cytology (IAC) introduced the Yokohama system for breast FNAC. This system categorizes aspirates into five interpretive groups: C1 (inadequate/insufficient); C2 (benign); C3 (atypical); C4 (suspicious for malignancy); C5 (malignant). These categories help streamline reporting practices and facilitate correlation with histopathology and clinical findings [4,5].

Parallel to this, Masood proposed a cytological scoring method for palpable breast lesions that evaluates architectural patterns and nuclear characteristics, including pleomorphism, anisonucleosis, chromatin quality, nucleoli, and the presence of myoepithelial cells. Based on these criteria, aspirates are placed into four diagnostic groups [6]. Subsequent work by Nandini et al. suggested that rearranging the scoring thresholds for the first two categories improves the diagnostic precision of the system [7]. This modification resulted in the Modified Masood Scoring Index (MMSI), which has since been explored to enhance classification accuracy in breast cytology [8,9].

Aim

To evaluate the role of the IAC Yokohama system and the MMSI in the cytological grading of breast lesions.

Objective

Our objective was to evaluate the diagnostic accuracy of the IAC Yokohama system and the MMSI in palpable breast lesions, as to which of these grading systems is better in terms of clinico-pathological correlation, inclusiveness of all breast lesions, and better understanding of tumor patterns.

## Materials and methods

Study design

This is a retrospective comparison study conducted in the Department of Pathology, Atal Bihari Vajpayee Government Medical College, Vidisha, Madhya Pradesh. A total of 535 patients with a breast lump were referred for FNAC, of whom 174 (32.52%) patients were followed up with histopathological examination (HPE) in our department between January 2023 and July 2024 (duration of 18 months). The cytopathology diagnosis was graded according to both the Yokohama and MMSI grading systems and then compared with respect to the histopathological diagnosis.

Inclusion criteria included all patients who came for cytomorphological examination and also went for HPE after surgery.

Exclusion criteria included patients with a prior history of chemotherapy or radiotherapy for any breast tumor.

After applying the inclusion criteria, cases diagnosed with both cytopathology and histopathology were selected, and slides were retrieved. The cytology slides were reviewed by trained cytopathologists in a single-blind manner. These slides were first reviewed and graded according to the Yokohama system by cytopathologist A, and then the same slides were reviewed and graded by cytopathologist B. The grading by both of them was compared. Then, again, the same slides were reviewed and scored according to the MMSI system by cytopathologist A and then by cytopathologist B. Scores given by both of them were compared.

A total of 535 FNAC requisitions for palpable breast lesions were received in the study period, and 174 of these patients subsequently underwent HPE. After assessing the cytopathology diagnosis and scoring, corresponding histopathology slides of the included cases were retrieved and reviewed by histopathologist A and then by histopathologist B, and were categorized as benign and malignant independently. Categories of both histopathologists were compared. Both cytopathologists and histopathologists were kept unaware of each other's findings.

Statistical analysis

Both cytopathology grading systems, i.e., the Yokohama and MMSI systems, were compared with histopathological diagnosis and categories taken as standard. The sensitivity, specificity, positive predictive value (PPV), and negative predictive value (NPV) of both diagnostic classification systems were determined using histopathology as the reference standard. Additionally, the diagnostic accuracy of each system was assessed individually. For the IAC Yokohama system, the risk of malignancy (ROM) for each category was calculated by dividing the number of cases with confirmed malignant lesions by the total number of cases with available histopathological diagnosis in that category.

## Results

**Figure 1:**
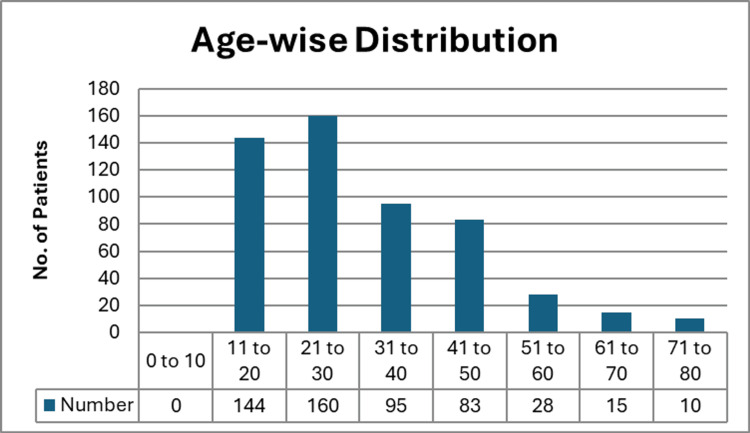
Graph showing the age distribution of the patients.

We received a total of 535 requisitions for FNAC of palpable breast lesions, out of which 174 patients were followed up with HPE. The maximum number of patients was in the 21-30-year age group, followed by the 11-20-year age group (Figure 1).

The cytological findings from FNAC were evaluated and classified according to the IAC Yokohama system (Table 1).

**Table 1:** Classification of breast lesions based on the International Academy of Cytology (IAC) Yokohama classification.

Yokohama grading	Number	Percentage
I. Inadequate	5	0.93%
II. Benign	410	76.64%
III. Atypical	42	7.85%
IV. Suspicious for malignancy	13	2.42%
V. Malignant	65	12.14%

Of the total cases, five (0.93%) were classified as inadequate (category 1), 410 (76.64%) as benign (category 2), 42 (7.85%) as atypical (category 3), 13 (2.42%) as suspicious for malignancy (category 4), and 65 (12.14%) as malignant (category 5).

Out of them, 174 cases were followed up with HPE. HPE findings of all cases were divided based on benign and malignant diagnosis, and ROM was noted for each of the grades (Table 2).

**Table 2:** Cases with assigned Yokohama grades and their correlation with HPE findings. HPE: histopathological examination; ROM: risk of malignancy.

Yokohama grading	Numbers	Numbers with HPE	HPE finding		ROM (%)
			Benign	Malignant	
I. Inadequate	5	1	1	0	0
II. Benign	410	135	134	1	0.74
III. Atypical	42	13	10	3	23.07
IV. Suspicious for malignancy	13	4	1	3	75
V. Malignant	65	21	0	21	100

**Figure 2:**
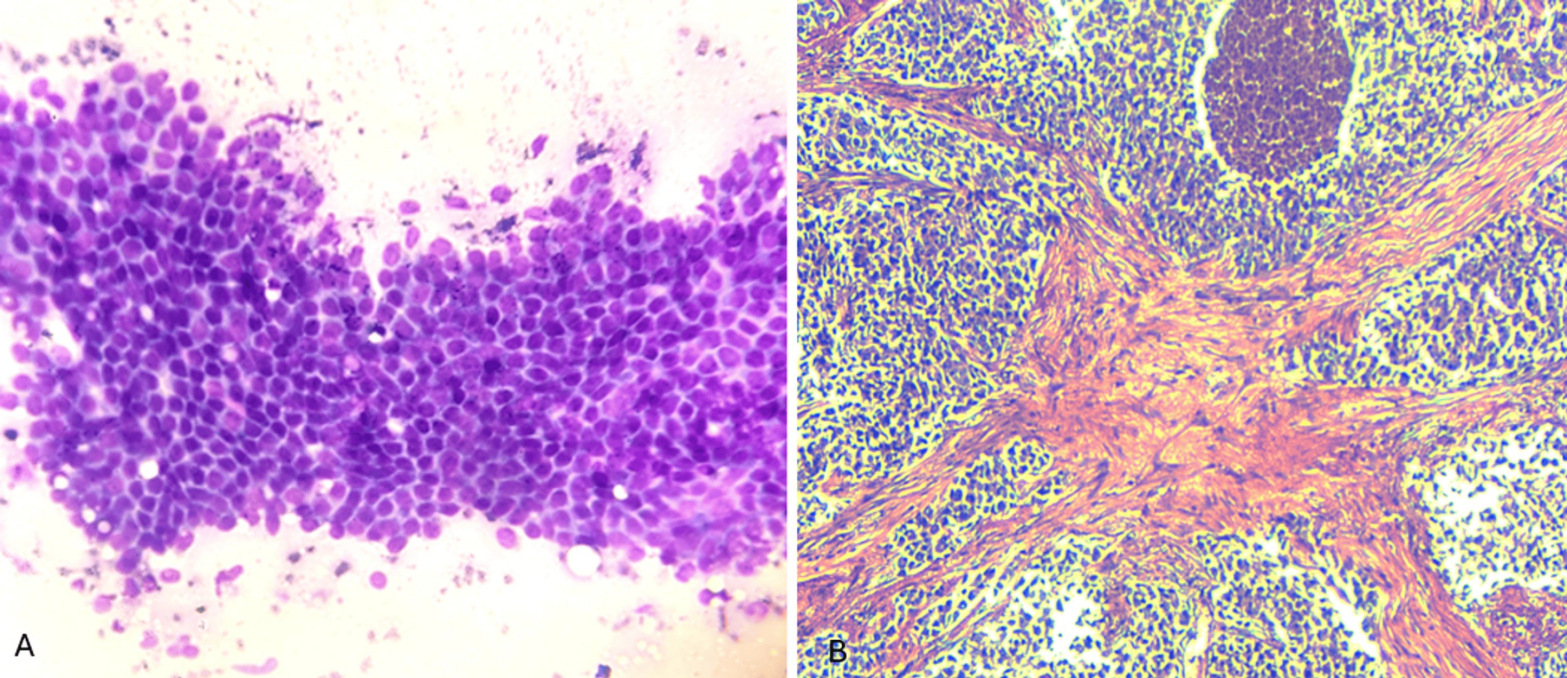
(A) Fine needle aspiration smear showing fibroadenoma, Yokohama category C2 (benign) (40x, Giemsa). (B) Histopathological section showing infiltrating ductal carcinoma (10x, H&E).

Out of five cases in category 1, only one was followed up with HPE, which turned out to be benign. A total of 135 out of 410 cases underwent HPE, in which 134 remained benign, showing good correlation with FNAC; however, one case turned out to be malignant (Figure 2).

**Figure 3:**
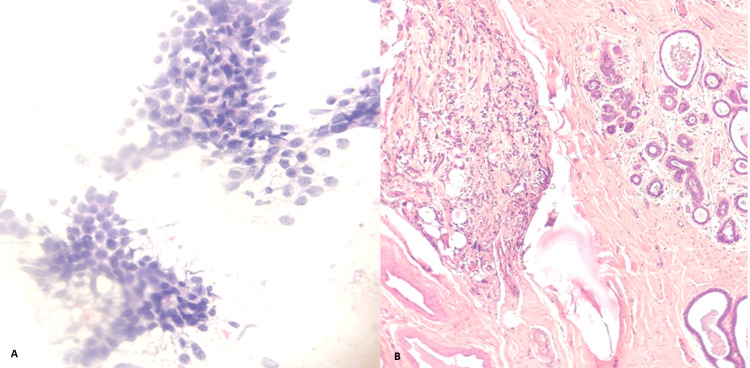
(A) Fine needle aspiration smear showing atypical ductal hyperplasia, Yokohama category C3 (atypical) (40x, Pap). (B) Corresponding breast tissue on histopathological examination showing ductal carcinoma in situ (10x, H&E).

A total of 13 out of 42 cases were followed by HPE, in which 10 were benign, and three cases turned out to be malignant (Figure 3).

**Figure 4:**
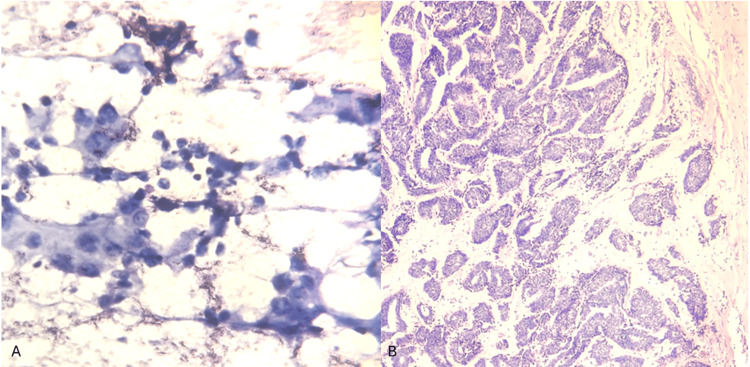
(A) Fine needle aspiration smear showing ductal carcinoma, Yokohama category C5 (malignant) (40x, Pap). (B) Corresponding breast tissue showing infiltrating ductal carcinoma on histopathological examination (10x, H&E).

Out of four cases that were suspicious for malignancy, three cases were confirmed as malignant on HPE. The rest of the 21 cases were malignant on both cytology and histopathology (Figure 4).

Statistical formulas for sensitivity, specificity, PPV, and NPV were applied, taking histopathological diagnosis as the confirmatory diagnosis. Yokohama categories I and II were considered benign, whereas categories III, IV, and V were considered malignant (Table 3).

**Table 3:** Values of different statistical tools. TP: true positive; TN: true negative; FP: false positive; FN: false negative.

Statistical tool	Formula	Percentage value
Sensitivity	TP/TP+FN	96.42%
Specificity	TN/TN+FP	92.46
Positive predictive value	TP/TP+FP	71.05%
Negative predictive value	TN/TN+FN	99.26%
Diagnostic accuracy	TP+TN/TP+TN+FN+FP	93.10%

The same cases were given scores as per cytological findings according to the MMSI (Tables 4, 5) [6,7].

**Table 4:** Scoring system for the interpretation of FNAC (Modified Masood Scoring Index). Modified Masood Scoring Index [6,7]. FNAC: fine needle aspiration cytology.

Cellular arrangement	Cellular pleomorphism	Myoepithelial cells	Anisonucleosis	Nucleoli	Chromatin clumping	Score
Monolayer	Absent	Many	Absent	Absent	Absent	1
Nuclear overlapping	Mild	Moderate	Mild	Micro nucleoli	Rare	2
Clustering	Moderate	Few	Moderate	Micro nucleoli and/or macro nucleoli	Occasional	3
Loss of cohesion	Conspicuous	Absent	Conspicuous	Predominantly macro nucleoli	Frequent	4

**Table 5:** MMSI categories based on scores from Table 4. Modified Masood Scoring Index (MMSI) [6,7].

Category	Score
Category I: Non-proliferative breast disease	6-8
Category II: Proliferative breast disease without atypia	9-14
Category III: Proliferative breast disease with atypia	15-18
Category IV: Carcinoma in-situ/carcinoma	19-24

Then, MMSI categories were allotted based on scores (Table 6).

**Table 6:** Classification of breast lesions based on the Modified Masood Scoring Index (MMSI).

MMSI grade	Numbers
Inconclusive	70
I: Non-proliferative breast disease	354
II: Proliferative breast disease without atypia	31
III: Proliferative breast disease with atypia	13
IV: Carcinoma in situ/carcinoma	67

Around 70 cases could not be given MMSI scores as the latter is based on cellular and nuclear morphology. Out of them, 15 cases were followed up with HPE, and among them, 13 were benign, including galactocele, fibroadenoma, granulomatous mastitis, and lactating adenoma. The remaining two cases turned out to be malignant, both of them were ductal carcinoma in situ with infiltrating ductal carcinoma (Table 7).

**Table 7:** Cytopathological diagnosis of inconclusive cases in MMSI, but graded as benign in the Yokohama system. MMSI: Modified Masood Scoring Index; HPE: histopathological examination; DCIS: ductal carcinoma in situ; EIC: epidermoid inclusion cyst.

No. of cases (out of 15)	Cytology diagnosis	Yokohama	HPE diagnosis
2	Galactocele	C2. Benign	Fibroadenoma
5	Galactocele	C2. Benign	Galactocele with lactating adenoma
1	Abscess	C2. Benign	Galactocele
2	Fat necrosis	C2. Benign	DCIS with infiltrating ductal carcinoma
2	Infected EIC	C2. Benign	Granulomatous mastitis
3	Galactocele	C2. Benign	Galactocele

Among the remaining 465 cases, the maximum number of patients were found to belong to category I (non-proliferative breast disease), which included 354 patients, followed by 67 cases in category IV (carcinoma in situ/carcinoma). Categories II and III included 31 and 13 cases, respectively. A total of 159 out of 465 patients were followed up with a histopathological diagnosis. A total of 119 out of 120 cases of category I (non-proliferative breast disease) were consistent with benign histopathological findings. A similar finding was also true for category II (proliferative breast disease without atypia), where 13 out of 14 were benign on HPE. Two out of three cases in category III (proliferative breast disease with atypia) were consistent with malignant histopathology (Table 8).

**Table 8:** Number of cases in MMSI categories and their correlation with HPE findings. MMSI: Modified Masood Scoring Index; HPE: histopathological examination.

MMSI category	Numbers	Numbers with HPE done	HPE	
			Benign	Malignant
I: Non-proliferative breast disease	354	120	119	1
II: Proliferative breast disease without atypia	31	14	13	1
III: Proliferative breast disease with atypia	13	3	1	2
IV: Carcinoma in situ/carcinoma	67	22	0	22

The remaining 22 cases were malignant on both cytopathology and histopathology. As mentioned earlier, two cases were reported as inconclusive in MMSI but were found to be malignant in histopathology and were thus excluded.

Categories I and II of MMSI were considered benign, whereas categories III and IV were considered malignant on histopathology (Table 9).

**Table 9:** Values of different statistical tools for MMSI. MMSI: Modified Masood Scoring Index; TP: true positive; TN: true negative; FP: false positive; FN: false negative.

Statistical tool	Formula	Percentage value
Sensitivity	TP/TP+FN	92.31%
Specificity	TN/TN+FP	99.25%
Positive predictive value	TP/TP+FP	96%
Negative predictive value	TN/TN+FN	98.51%
Diagnostic accuracy	TP+TN/TP+TN+FN+FP	98.11%

Statistical formulas of sensitivity, specificity, PPV, and NPV were calculated in both systems for comparing the cytology with histopathology findings. McNemar's testing for binary comparison of test criteria was applied in comparison to the histopathological findings, and the p-value was calculated.

After applying both cytological systems and comparing with the histopathological diagnosis, statistical tests were compared on both diagnostic criteria (Table 10).

**Table 10:** Comparison between the Yokohama grading system and the MMSI system. MMSI: Modified Masood Scoring Index.

Parameters	Yokohama	MMSI
Sensitivity	96.42%	92.31%
Specificity	92.46%	99.25%
Positive predictive value	71.05%	96%
Negative predictive value	99.26%	98.51%
Diagnostic accuracy	93.10%	98.11%

The Yokohama system showed a sensitivity of 96.42%, a specificity of 92.46%, a PPV of 71.05%, and an NPV of 99.26%. Whereas, the MMSI system showed a sensitivity of 92.31%, a specificity of 99.25%, a PPV of 96%, and an NPV of 98.51%. Overall, MMSI showed better diagnostic accuracy of 98.11% than 93.10% in the Yokohama system. McNemar’s test for paired binary outcomes showed that MMSI had significantly better agreement (p-value = 1.0) with histopathology than the Yokohama system (p-value = 0.0063).

## Discussion

Breast lesions represent one of the most prevalent conditions among females, and the increasing incidence of breast malignancies worldwide, particularly cases of triple-negative breast cancer, remains a significant public health concern. Presently, the assessment of a breast mass is best conducted through the triple assessment method, which encompasses clinical examination, radiological imaging, and pathological evaluation to optimize diagnostic accuracy and patient care [5]. Ancillary methods such as core needle biopsy, immunocytochemistry, and molecular diagnostics further contribute to diagnostic precision, particularly in situations where cytology specimens are the sole material available, such as in locally advanced or metastatic breast cancer.

In the present study, 535 breast FNAC samples were categorized using both the IAC Yokohama system and the MMSI, of which 174 cases had histopathological correlation. The comparison of these two widely used systems offers valuable insights into their diagnostic strengths, limitations, and practical applicability.

Performance of the IAC Yokohama system

The majority of cases in this study were classified as benign (category II) under the Yokohama system, which was consistent with global and Indian data showing benign lesions as the most common FNAC category in routine practice [2,3,10,11]. Among 535 cases, the distribution pattern in the present study, i.e., 5 (0.93%) inadequate, 410 (76.64%) benign, 42 (7.85%) atypical, 13 (2.42%) suspicious, and 65 (12.14%) malignant, closely parallels other large series where benign lesions dominated breast FNAC reporting. Among 174 cyto-histologically correlated cases, the ROM showed a clear rising trend in accordance with the IAC definitions (Table 11).

**Table 11:** Comparison of risk of malignancy (ROM) between different studies and the present study. IAC: International Academy of Cytology.

ROM (%) IAC Yokohama	Montezuma et al. [10]	Ahuja et al. [3]	Wong et al. [11]	Bhuyan et al. [2]	Current study
Inadequate	4.8	5	2.6	12.5	0
Benign	1.4	1.5	1.7	1.8	0.74
Atypical	13	17.4	15.7	20	23.07
Suspicious for malignancy	97.1	81.8	84.6	90.4	75
Malignant	100	100	99.5	97.5	100

This gradient is remarkably consistent with the ROM ranges provided in the IAC Atlas by Field et al. (benign ROM = 1.4-2.3%; atypical = 13-15.7%; suspicious = 84-97%) [4]. The perfect 100% ROM in the malignant category reinforces the high specificity of classic malignant cytomorphological features.

The diagnostic performance of the Yokohama system in our study closely matched that reported by Ahuja et al. (sensitivity up to 97.2% and accuracy of 96.4%) and Sreevidyalatha et al. (accuracy of 88.96%) [1,3]. This consistency supports the reproducibility of Yokohama categories across diverse settings (Table 12).

**Table 12:** Comparison of the diagnostic performance of the Yokohama system across different studies and the present study. PPV: positive predictive value; NPV: negative predictive value; IAC: International Academy of Cytology.

Study	Sensitivity (%)	Specificity (%)	PPV (%)	NPV (%)	Diagnostic accuracy (%)
Present study (2025)	96.42	92.46	71.05	99.26	93.10
Ahuja & Malviya (2021) [3]	97.2	100	100	95.6	96.4
Sreevidyalatha et al. (2023) [1]	86.75	97.32	99.19	66.06	88.96
Field et al. [4], IAC Yokohama Atlas [5] (Global Data Range)	90–99	95–100	~100	90–98	Up to 96.2

The Yokohama system's strength lies in its explicit linkage between each category, ROM, and recommended clinical management, enabling seamless integration with the “triple test” approach. This structured, standardized communication was a major goal when the system was developed by the IAC.

Performance of the MMSI

The MMSI categorizes breast lesions based on a six-parameter scoring system and is especially valuable for proliferative breast disease (PBD). This study’s distribution was typical of MMSI applications, with most cases falling into the non-proliferative breast disease category (75%). These values align with published MMSI studies. Agarwal et al. [12] noted improved categorization of proliferative lesions and high concordance in atypical categories, and Yadav et al. [9] reported excellent concordance in malignant categories. In our study, although the MMSI performed well, its sensitivity and NPV were slightly lower than those of the Yokohama system (Table 13).

**Table 13:** Comparison of the diagnostic performance of the MMSI across different studies and the present study. PPV: positive predictive value; NPV: negative predictive value; MMSI: Modified Masood Scoring Index.

Study	Sensitivity (%)	Specificity (%)	PPV (%)	NPV (%)	Diagnostic accuracy (%)
Present study	92.31	99.25	96	98.51	98.11
Agarwal et al. (2019) [12]	95	100	100	97.6	98.3
Yadav et al. (2023) [9]	95.7	88.4	93.4	92.3	93.1
Nandini et al. (2011) [7]	96	87	92.6	98	92

Why the Yokohama system is superior to the MMSI

Despite the MMSI’s strengths, especially in evaluating early proliferative lesions, the IAC Yokohama system demonstrated superior diagnostic value in this study for several reasons. First, clearer definitions and a ROM-based structure; the Yokohama system’s tiered system links diagnostic categories to quantifiable ROM values and management pathways, which is absent in MMSI. This inherently improves clinical decision-making and reduces ambiguity in reporting [13]. The Yokohama system outperformed the MMSI in both sensitivity (96.42% vs. 92.31%) and NPV (99.26% vs. 98.51%). Higher sensitivity reduces false negatives, which is crucial for the detection of early cases. A key advantage of the Yokohama system is the creation of distinct “Atypical” and “Suspicious” categories, each with validated ROM ranges. The MMSI clusters multiple borderline lesions into numerical brackets, which may blur their risk stratification [14]. While the MMSI is validated mainly in South Asian centers, the Yokohama system has undergone extensive international validation across high- and low-resource settings. Its adoption is now widespread due to simplicity, reproducibility, and direct clinical relevance [15].

The MMSI requires scoring six point-based cytological parameters. In contrast, the Yokohama system uses broader, morphology-driven categories with fewer subjective elements. The MMSI offers useful cytomorphologic detail and may improve internal grading, but the Yokohama system is often more practical for routine breast cytology because it provides a standardized, category-based framework with clearer risk-of-malignancy stratification and easier clinical communication [8-10]. In borderline and equivocal lesions, the Yokohama system reduces ambiguity by allowing explicit reporting of inadequate, atypical, suspicious, and malignant categories rather than forcing cases into a numerical score. This improves reproducibility, facilitates clinicopathological correlation, and aligns cytology more closely with histopathologic follow-up and management decisions [11]. Thus, the MMSI remains a valuable scoring tool, whereas the Yokohama system is better suited to standardized reporting in daily practice. Together, these factors may explain the comparatively better performance of the Yokohama system in our study [4,16].

MMSI: Context where it remains valuable

Although the Yokohama system is superior for routine cytology reporting, the MMSI has distinct strengths in (a) the detailed evaluation of PBD, (b) research on the progression from atypia to carcinoma, (c) cases in which subtle nuclear changes require quantification, and (d) academic settings focused on PBD pathology. Thus, the MMSI is a useful complementary tool, though less suitable as a universal reporting system [6,12,17].

Strengths of the study

The strengths of this study include direct comparison of two established breast cytology reporting systems in the same cohort, histopathological correlation as the reference standard, and its reflection of routine tertiary care diagnostic practice, thereby enhancing the clinical relevance and applicability of the findings.

Limitations of the study

This was a retrospective study conducted over a period of 18 months. Only one-third of cases (174/535) had histopathological correlation, which may underestimate ROM for benign categories. The MMSI scoring remains partly subjective to the individual pathologists and seems to be time-intensive.

## Conclusions

This comparative study highlights that while both the MMSI and the IAC Yokohama system are effective cytological tools for breast FNAC, the Yokohama system demonstrates greater overall suitability. Although the MMSI showed stronger statistical parameters in certain aspects and remains valuable for detailed evaluation of proliferative lesions, it lacked scoring in some cases where the Yokohama system provided clear categorization. The Yokohama system offers superior diagnostic accuracy, standardized risk stratification, and enhanced clinical communication, giving surgeons better guidance in patient management. Its ROM-based, globally validated framework makes it more reliable and practical for universal adoption as the primary reporting system in routine breast cytology.
